# Pye's Surgical Handicraft

**Published:** 1892-09

**Authors:** 


*Pye's Surgical Handicraft.* Third Edition. Revised and Edited by T. H. R. CROWLE, F.R.C.S. Pp. xx., 570. Bristol: JOHN WRIGHT & Co. 1891.

This well-known text-book for house-surgeons has been brought well up to date in the present edition, and will doubtless maintain its reputation.

We notice a few blemishes. On page 9 is a very absurd and incorrect diagram intended to show the position of the principal arteries, but which could only serve to mislead the unwary. In the treatment of profuse hemorrhage, no mention is made of transfusion of saline fluid. In speaking of the dressing of wounds, great reliance is placed upon water dressings and carbolic oil—these are even spoken of as the best dressings for scalp wounds and for wounds into joints!

The book is, upon the whole, a trustworthy and practical guide in all cases of emergency which come before the house-surgeon or dresser.

